# Fast and reliable detection of toxic *Crotalaria spectabilis* Roth. in *Thunbergia laurifolia* Lindl. herbal products using DNA barcoding coupled with HRM analysis

**DOI:** 10.1186/s12906-015-0692-6

**Published:** 2015-05-30

**Authors:** Sahachat Singtonat, Maslin Osathanunkul

**Affiliations:** Department of Biology, Faculty of Science, Chiang Mai University, Chiang Mai, 50200 Thailand

## Abstract

**Background:**

Nowadays, medicinal plants are used as a popular alternative to synthetic drugs. Many medicinal plant products have now been commercialized throughout various markets. These products are commonly sold in processed or modified forms such as powders, dried material and capsules, making it almost impossible to accurately identify the constituent species. The herbal plant known as ‘Rang Chuet’ in Thai has been widely used as remedies for various ailments. However, two medicinal plants species, *Thunbergia laurifolia* and *Crotalaria spectabilis* share this name. Duo to the similarity in nomenclature, the commercial products labeled as ‘Rang Chuet’ could be any of them. Recently, the evidence of hepatotoxic effects linked to use of *C. spectabilis* were reported and is now seriously concern. There is a need to find an approach that could help with species identification of these herbal products to ensure the safety and efficacy of the herbal drug.

**Methods:**

Here DNA barcoding was used in combination with High Resolution Melting analysis (Bar-HRM) to authenticate *T. laurifolia* species. Four DNA barcodes including *matK*, *rbcL*, *rpoC* and *trnL* were selected for use in primers design for HRM analysis to produce standard melting profiles of the selected species. Commercial products labeled as ‘Rang Chuet’ were purchased from Thai markets and authentication by HRM analyses.

**Results:**

Melting data from the HRM assay using the designed primers showed that the two ‘Rang Chuet’ species could easily be distinguished from each other. The melting profiles of the all four region amplicons of each species are clearly separated in all three replicates. The method was then applied to authenticate products in powdered form. HRM curves of all ten test samples indicated that three of the tested products did not only contain the *T. laurifolia* species.

**Conclusion:**

The herbal drugs derived from different plants must be distinguished from each other even they share the same vernacular name. The Bar-HRM method developed here proved useful in the identification and authentication of herbal species in processed samples. In the future, species authentication through Bar-HRM could be used to promote consumer trust, as well as raising the quality of herbal products.

**Electronic supplementary material:**

The online version of this article (doi:10.1186/s12906-015-0692-6) contains supplementary material, which is available to authorized users.

## Background

### Herbal medicines

Natural products from plants have played a considerable role in the way of life of people around the world since ancient times. Plant products have been consumed as food and used as medicinal remedies. An enormous number of scientific reports highlight the benefits of using medicinal plants and herbs as an alternative to modern synthetic drugs [1]. It is clear that medicinal plants are a popular alternative to synthetic drugs. According to the World Health Organization [2], over 70% of the world’s population in developing countries uses herbal products. Many medicinal plant products have now been commercialized. These products are commonly sold in processed or modified forms such as powders, dried material, tablets, capsules and tea bags, making it almost impossible to accurately identify the constituent species [3–5]. Because of this, consumer safety could be a concern. Misidentification of the constituent plants may lead to the inclusion of undesirable, unrelated species, with a potential health risk to the end users. Substitution of the product’s ingredients either intentionally or inadvertently can have negative effect on both consumers and producers. Because of these identification issues, any measures that may aide in the identification of herbal products would be beneficial. Many species from the Acanthaceae family are considered in Thailand to have health benefits, several species (*Acanthus ebracteatus*, *Andrographis paniculata*, *Rhinacanthus nasutus* and *Thunbergia laurifolia*) are now included on the Thai National List of Essentials Medicine (NLEM; Thailand). These Acanthaceae species are commonly used in Thai household as remedies for various ailments and thus are regularly sold on the markets throughout Thailand. There is a need to find an approach that could help with the quality control of these herbal products to ensure both the satisfaction and safety of consumers.

*Thunbergia laurifolia* is one of common Thai medicinal plant with various used such as antipyretic, detoxification against insecticides, alcoholic and metallic poisons. *T. laurifolia* commonly known in Thai as ‘Rang Chuet’ has been used in Thailand as a natural remedy for decades. Commercial products of ‘Rang Chuet’ in tea, capsule, and powder forms in herbal markets are claimed to have beneficial effects on human health. However, confusion has arisen because of the similarity in the vernacular names of the plants. In Thailand, there are at least three species are being called ‘Rang Chuet’, one of these is *Crotalaria spectabilis*. There are several documentations reported that seeds and leaves of *C. spectabilis* contain pyrrolizidine alkaloids which causes Hepatotoxicity in humans and mammals [6–10]. Due to the fact that, Rang Chuet was immensely sold in Thailand local markets and used as household remedy in form of processed products. Therefore it is almost impossible for consumer to know exactly which ‘Rang Chuet’ products they are buying so this could be a real big issue and is now seriously concern.

### Molecular species identification

Recently, many works have been focused on authentication or detection of species substitution of herbal products. Molecular techniques like RFLP and RAPD were developed for Rang Chuet identification [4, 5]. Both RFLP and RAPD technique showed a potential in discrimination of the two Rang Chuet (*T. laurifolia* and *C. spectabilis*) species but the disadvantage of RFLP is not only relatively time consuming but the RFLP also requires a large amount of sample, although RAPD technique is fast but many [11–14] show unstable results and sometimes unreproducible.

In the past decade, DNA barcoding (short DNA sequence) is proved to be useful for identifying and categorizing species [15]. To date, several Rang Chuet barcodes from various plastid genome regions including *matK*, *rpl*16, *rps*16, *trnL* and *rbcL* were produced [5, 14]. Although DNA barcoding is proven useful for species-level identification of plants [16, 17], there are some limitations to the technique. It is costly and time-consuming, and not easy to apply routinely in developing countries due to financial constraints and limited availability of perishable chemicals and consumables. This leads us in searching for new fast, reliable, less time consuming and inexpensive method for species identification and authenticating of medicinal plants. Here we applied DNA barcoding with high resolution melting (Bar-HRM) analysis for species identification and authentication of ‘Rang Chuet’ products sold on Thai markets. The use of Bar-HRM for taxonomic identification and the detection of adulteration in food and agriculture products has been reported recently [18–20]. In this study we evaluate whether the technique is equally useful for species discrimination in constituents of Thai folk medicinal plant.

## Materials and methods

### Primers used for HRM analysis

Sequences of the plastid DNA regions, *matK, rbcL*, *rpoC* and *trnL* of selected medicinal plants from the family Acanthaceae (*Thunbergia* spp.) and Fabaceae (*Crotalaria* spp.) were extracted from GenBank (at the end of September 2013) using the key phrases “the name of locus” and “the name of species” in the annotations. Generally, sequences obtained from public databases, including GenBank, are of low quality with no known associated herbarium vouchers. For this reason, all of the sequences were subjected to critical evaluation and any low-quality sequences were removed. After processing, multiple alignments were made from the selected sequences using MEGA6 [21] and variable characters were calculated for the design of primers to be used for high resolution melting (HRM) analysis. Two main criteria were considered in order to obtain successful results in the HRM analysis: (i) the primer pair should generate a PCR product not exceeding 300 bp, (ii) the primer pairs should cover enough variable sites to enable discrimination among the tested species and any other variation site from sequence of conspecifics.

### Plant samples and DNA isolation

Both fresh and dried samples were included in this study (Table 1). Two ‘Rang Chuet’ herb species (*T. laurifolia* and *C. spectabilis*) were the main focus of the study. Fresh specimens of these species were collected from areas in Chiang Mai province, Thailand. Dried plant tissues for DNA extraction were kindly provided by Queen Sirikit Botanic Garden (QSBG). The plant material was ground with liquid nitrogen, and then used for DNA extraction with the Nucleospin Plant® II kit (Macherey-Nagel, Germany) following the manufacturer’s instruction. DNA concentrations of all samples were equally adjusted (20 ng/μL). The DNA was stored at−20° C for further use.

**Table 1:** Plants species and commercial products included in this study

Species/Type	Abbreviation	Source	Sample type
*Thunbergia laurifolia*	T1	Materia Medica garden	Fresh
		Faculty of Pharmacy, Chiang Mai University	
*Thunbergia laurifolia*	T2	Department of Biology	Fresh
		Faculty of Science, Chiang Mai University	
*Thunbergia laurifolia*	T3	Queen Sirikit Botanical Garden	Dry
		Mae Rim, Chiang Mai (voucher number 46323)	
*Thunbergia laurifolia*	T4	Queen Sirikit Botanical Garden	Dry
		Mae Rim, Chiang Mai (voucher number 59427)	
*Crotalaria spectabilis*	C1	Materia Medica garden	Fresh
		Faculty of Pharmacy, Chiang Mai University	
*Crotalaria spectabilis*	C2	Materia Medica garden	Fresh
		Faculty of Pharmacy, Chiang Mai University	
*Crotalaria spectabilis*	C3	CMU Biology Garden	Fresh
		Faculty of Science, Chiang Mai University	
Commercial CN-C	COM1	Chiang Mai	Capsule
Commercial TT-C	COM2	Chiang Mai	Capsule
Commercial HBO-C	COM3	Chiang Mai	Capsule
Commercial HBO-T	COM4	Chiang Mai	Tea bag
Commercial APB-P	COM5	Lamphun	Powder
Commercial NK-L	COM6	Lamphun	Dried leaf
Commercial GT-L	COM7	Pa Yao	Dried leaf
Commercial OTOP-T	COM8	Pa Yao	Tea bag
Commercial RTN-S1	COM9	Pa Yao	Dried bark
Commercial RTN-S2	COM10	Pa Yao	Dried bark

### Real-time PCR amplification and high resolution melting (HRM) analysis

To determine the characteristic melting temperature (T_m_) for each sample that could be used to distinguish the two different ‘Rang Chuet’ medicinal plants, PCR amplification, DNA melting, and end point fluorescence level acquiring PCR amplifications were performed in a total volume of 20 μL on an Eco™ Real-Time PCR system (Illumina®, San Diego, USA). The reaction mixture contained 10 ng genomic DNA, 10 μL of MeltDoctor™ HRM Master Mix (Applied Biosystems, California, USA), 0.2 μL of 10 mM forward and reverse primers. The four pairs of candidate barcoding primers nucleotide composition are shown in Table 2. The real-time PCR reaction conditions are as following; an initial denaturing step at 95 °C for 5 min followed by 35 cycles of 95 °C for 30 s, 57 °C for 30 s, and 72 °C for 20 s. Subsequently, the PCR amplicons were denatured for HRM at 95 °C for 15 s, and then annealed at 50 °C for 15 s to form random DNA duplexes. Melting curves were generated after the last extension step. The temperature was increased from 60 to 95 °C at 0.1 °C/s. The melting curves were analyzed with the Eco™ software (version 4.0.7.0). After obtaining the suitable primers for the HRM in order to test the sensitivity of the developed method, real-time PCR and barcoding with HRM were carried out on standard samples, prepared by mixing fine powder of *T. laurifolia* with *C. spectabilis* in different proportions of 1, 3, 6, 12, 25, and 50 %. Real-time PCR amplification was performed as described earlier.

**Table 2:** Four primers used for HRM analysis and identification

Primer name	Nucleotide sequence (5’ to 3’)	T_a_ (°C)	Expected size (bp)
matK _F	CTTCTTATTTACGATTAACATCTTCT	57	160
matK _R	TTTCCTTGATATCGAACATAATG		
rbcL_F	GGTACATGGACAACTGTGTGGA	57	150
rbcL _R	ACAGAACCTTCTTCAAAAAGGTCTA		
rpoC_F	CCSATTGTATGGGAAATACTT	57	170
rpoC _R	CTTACAAACTAATGGATGTAA		
trnL_F	GAATCGACCGTTCAAGTATCC	57	150
trnL _R	TATAGGAAACCCATATTTGATCCAATC		

### Authenticating test of herbal products sold on Thai local markets

Ten herbal products labeled as ‘Rang Chuet’ were purchased for this study. All of the products were acquired in processed forms (Table 1). Total DNA was extracted from each sample and then used in HRM analysis in order to identify the characteristic melting temperature (T_m_).

## Results and discussion

### Data mining and primers used

The amplification of the four selected locus from two ‘Rang Chuet’ medicinal plant species (*T. laurifolia* and *C. spectabilis*) was performed using specific primers corresponding to the *matK*, *rbcL*, *rpoC* and *trnL* barcode region. All sequences of *Thunbergia* spp. were extracted from GenBank and the variable characters and average %GC content was calculated for all samples (Table 3). Data was present for most markers of the target species, except for *rpoC*. The total number of sequences retrieved for the respective markers were: *matK* 11 (7 species); *rbcL* 6 (6 species), *trnL* 27 (20 species). The absence of *rpoC* sequences for the target species was resolved by selecting random *rpoC* sequences from GenBank, which is supported by the high universality of *rpoC* [17, 22]. Two sequences of *C. spectabilis* were retrieved (*matK* and *trnL*).

**Table 3:** Characteristics of sequences and primers for high resolution melting analysis

Regions	*mat* *K*	*rbc* *L*	*rpoC*	*trn* *L*
Available species	5	6	5	19
Variable characters (%)	30.67	10.07	10.19	8.90
Conserved forward primer/total (%)	15/26 (57.69)	21/22 (95.45)	18/21 (85.71)	19/21 (90.48)
Conserved reverse primer/total (%)	19/23 (82.61)	25/25 (100)	19/21 (90.48)	24/27 (88.89)
Average %GC content	35.20	46.60	44.26	34.50

For *matK* 5, *rbcL* 6, *rpoC* 5 and *trnL* 19 sequences were deemed useful for further analysis (Table 4). An alignment of all useful sequences was made, and the primers flanking regions of each marker ranging from 150 to 170 bp were analyzed (Table 2). Reed and Wittwer [23] found that suitable length for HRM analysis should be 300 bp or less for optimal results.

**Table 4:** Sequences of four plastid regions (*matK, rbcL, rpoC* and *trnL*) were retrieved from GenBank (NCBI) for each of the species with accession number

Species	Regions			
	*trnL*	*matK*	*rbcL*	*rpoC*
*Crotalaria spectabilis*	HM208335	AB649973	-	-
*Thunbergia affinis*	AB817377	-	-	-
	EU315886			
*Thunbergia alata*	AF061820	HQ384512	HQ384878	-
	EU529130	AF531811		
	EU315887			
*Thunbergia angulata*	EU315888	-	-	-
*Thunbergia arnhemica*	EU315889	-	-	-
*Thunbergia atriplicifolia*	EU315890	-	-	-
*Thunbergia battiscombei*	EU315891	-	-	-
*Thunbergia capensis*	EU315892	AM234783	AM234783	-
*Thunbergia coccinea*	EU529131	HG004920	KF181493	-
*Thunbergia convolvulifolia*	EU315894	-	-	-
*Thunbergia dregeana*	EU315895	-	-	-
*Thunbergia erecta*	AF061821	AB649972	-	-
	JQ764614			
	EU529132			
	EU315896			
*Thunbergia fragrans*	U315897	-	-	-
*Thunbergia galpinii*	EU315898	-	-	-
*Thunbergia grandiflora*	EU315899	AB649971	JQ590086	-
		JQ586429		
		JQ586428		
		JQ586427		
*Thunbergia gregoryi*	EU315901	-	-	-
*Thunbergia guerkeana*	EU315901	-	-	-
*Thunbergia kirkii*	EU315902	-	-	-
*Thunbergia laurifolia*	-	AB649970	-	-
*Thunbergia mysorensis*	-	-	AY008828	-
*Thunbergia petersiana*	EU315904	-	-	-
*Thunbergia pondoensis*	EU315905	-	-	-
*Thunbergia togoensis*	EU315906	-	-	-
*Thunbergia usambarica*	-	-	L12596	-

Both the sequence length and the nucleotide variation within sequences influence the dissociation energy of the base pairs and result in different T_m_ values. The *matK* amplicon sequences were observed to have higher nucleotide variation than the amplicons of the other regions, at 30.67%. The relative nucleotide variation within amplicons was found to be as follows: *matK* > *rpoC* > *trnL* > *rbcL* (Table 3). The forward and reverse *matK* primers matched the consensus sequence of the target species at the binding sites in only 15 out of 26 sites (57.69%) and 19 out 23 of sites (82.61%), respectively (Table 3). High universality at the initial bases of the primer site is crucial for primer annealing and subsequent elongation initiation by the DNA polymerase. The *matK* locus is one of the most variable plastid coding regions and has high interspecific divergence and good discriminatory power. However, it can be difficult to amplify with the standard barcoding primers due to high substitution rates at the primer sites [24, 25]. The *rbcL, rpoC* and *trnL* primer pairs were expected to be a suitable primer for HRM analysis for discrimination between the tested plant species. These primers were nearly identical in base similarity to the mined consensus sequence (Table 3).

The average %GC content of amplicons was calculated in order to predict variation in melting curves for the different markers. *trnL* had the lowest average %GC content, with 34.50%, followed by *matK*, *rpoC* and *rbcL*, with 35.20, 41.85, 44.26 and 46.60% respectively (Table 3).

### Finding suitable primer pairs for discrimination between *T. laurifolia* and *C. spectabilis*

**Fig. 1:**
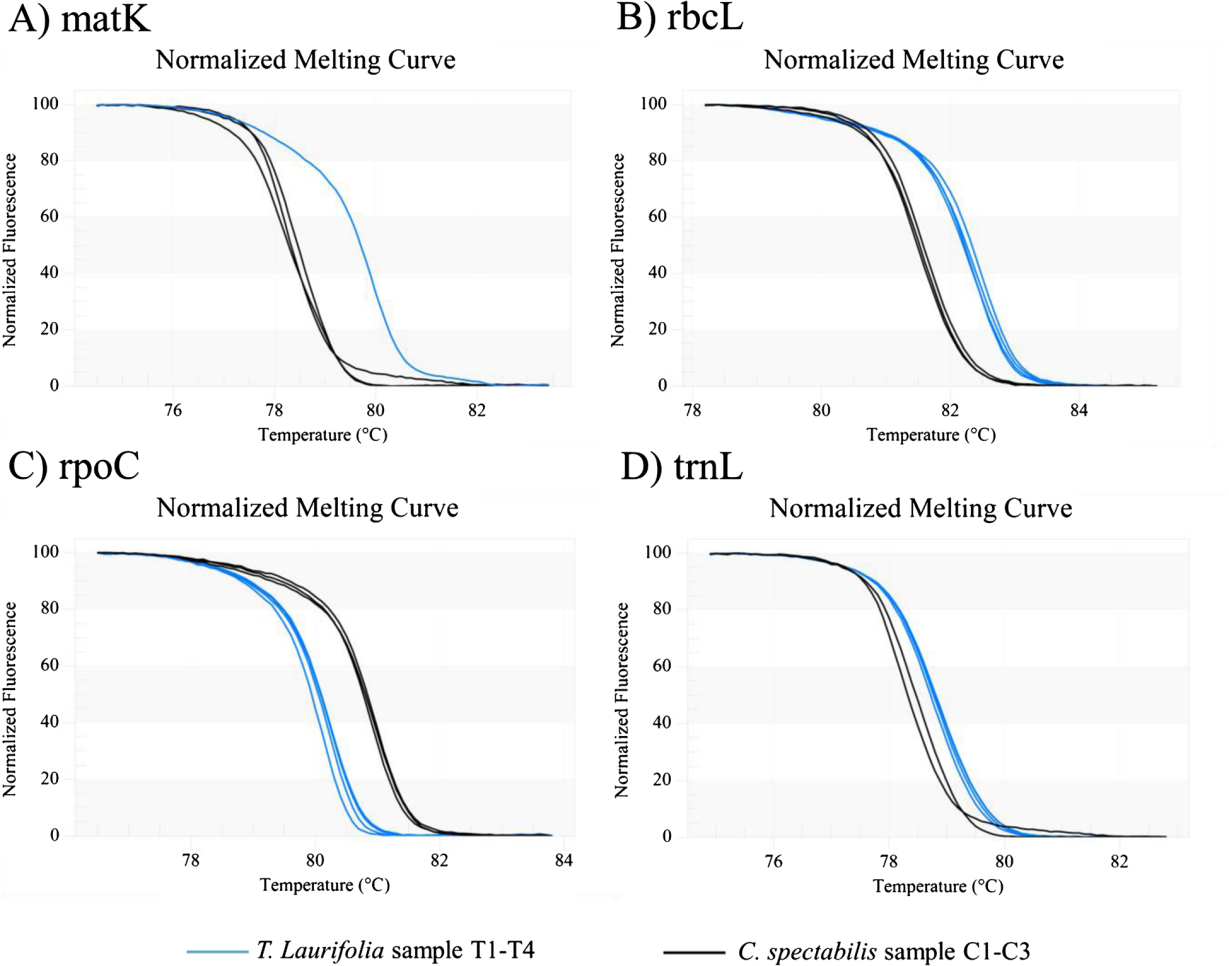
Melting curve profiles of amplicons obtained from each primer set. The normalized plot of each primer pair *matK* (**a**), *rbcL* (**b**), *rpoC* (**c**), and *trnL* (**d**) shows the differentiation of melting temperature (T_m_) of each amplicon from each species, generated by high resolution melting (HRM) analysis

The four primers sets were used for the amplification of DNA-fragments from all seven samples (two ‘Rang Chuet’ species), and the amplicons were analyzed using HRM to define T_m_. (Table 5). The expected length of amplified products from *matK*, *rbcL*, *rpoC*, and *trnL* are 160 bp, 150 bp, 170 bp, and 150 bp, respectively. The melting profiles of all amplicons are illustrated in Fig. 1a-1d. The analysis is presented by means of conventional derivative plots, which show that the T_m_ value of each species is represented by a peak. The samples of the two different species could be easily distinguished using HRM analysis with all four primer pairs. The melting profiles of seven samples of the two ‘Rang Chuet’ species (*T. laurifolia* and *C. spectabilis*) can be divided into two groups. All *T. laurifolia* samples (T1-T4) are grouped together and the other group contains all *C. spectabilis* samples (C1-C3). Although the four primer pairs tested could be used to discriminate *T. laurifolia* from *C. spectabilis*, the *rpoC* region was chosen for further analysis as it would help in demonstrating that Bar-HRM could work well as a sequencing-free method for plant identification. In addition, the *rpoC* region was used as an analytical target in HRM analysis has been shown to be effective for the detection and quantification of *Lens culinaris* and *Lathyrus clymenum* adulterations [18, 22].

**Table 5:** The values of melting temperature (°C) with standard deviations gaining form high resolution melting (HRM) analysis using *matK*, *rbcL*, *rpoC* and *trnL* primers of *T. laurifolia* and *C. spectabilis* species

Species	Abbreviation	Tm (^o^C)			
		*mat* *K*	*rbc* *L*	*rpo* *C*	*trn* *L*
*T. laurifolia*	T1	-	82.5 ± 0.07	80.2 ± 0.12	78.8 ± 0.07
*T. laurifolia*	T2	-	82.4 ± 0.14	80.1 ± 0.15	78.9 ± 0.07
*T. laurifolia*	T3	77.9 ± 0.35	82.3 ± 0.14	80.2 ± 0.00	78.8 ± 0.00
*T. laurifolia*	T4	-	82.4 ± 0.07	80.2 ± 0.07	78.8 ± 0.14
*C. spectabilis*	C1	75.6 ± 0.21	81.6 ± 0.00	80.9 ± 0.06	78.4 ± 0.14
*C. spectabilis*	C2	75.3 ± 0.07	81.6 ± 0.07	80.9 ± 0.06	-
*C. spectabilis*	C3	75.4 ± 0.21	81.5 ± 0.00	80.8 ± 0.00	78.2 ± 0.00

(-) No amplicons were generated

### Quantitative detection of *T. laurifolia* adulterants with Bar-HRM analysis

**Fig. 2:**
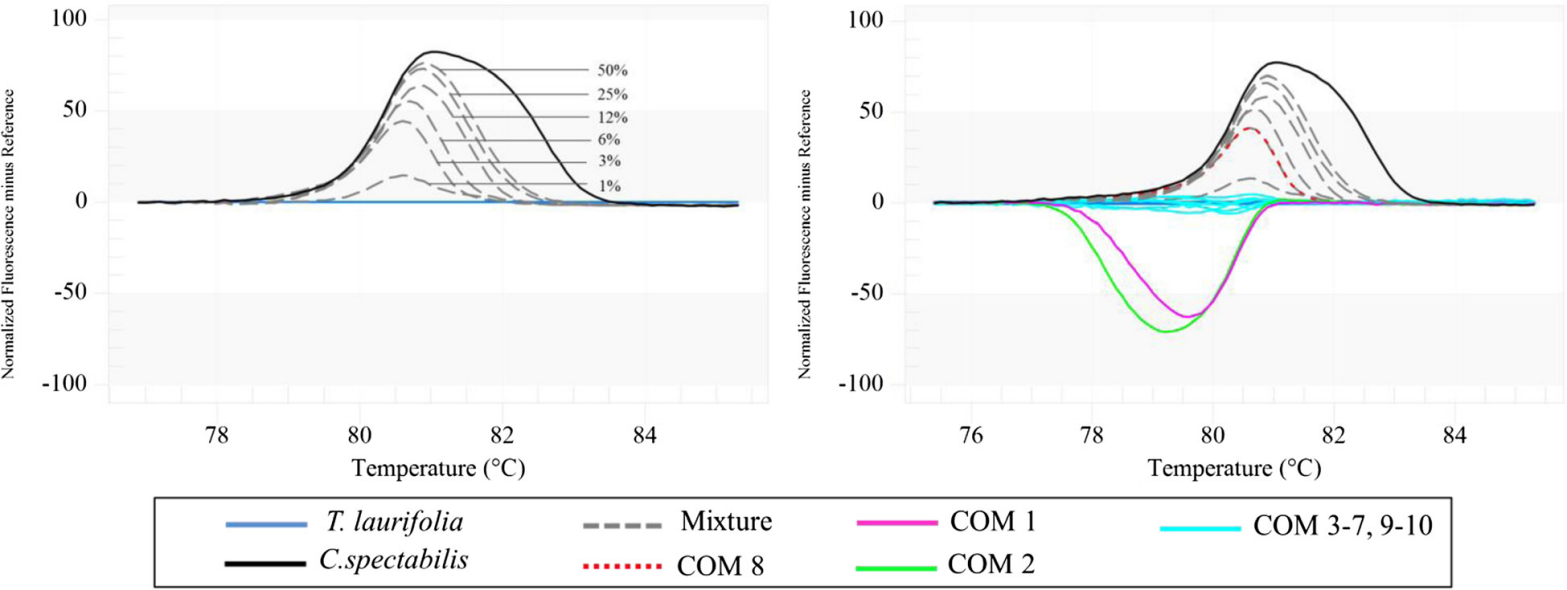
Melting curves obtained by high resolution melting analysis of the two ‘Rang Chuet’species. **a** Specific amplicons and applied to reference mixtures containing 50, 25, 12, 6, 3 and 1 % of *C. spectabilis* in *T. laurifolia.* **b** Difference graph of ten commercial herbal products using *T. laurifolia* as reference species. Data are from a single experiment

Detecting limit of adulteration in *T. laurifolia* products using the developed method with *rpoC* primers was tested. Figure 2a shows the results of the validation method with *T. laurifolia* spiked with *C. spectabilis* in different proportions. These results depict the analysis for one experiment as all three experiments gave similar results thus showing very good reproducibility. The process of the *T. laurifolia* amplicon dissociation reveals the level of contamination resulting from adulteration as the presence of increasing quantity of *C. spectabilis* into the *T. laurifolia* DNA alters the shape and shifts proportionally the melting curve, compared to the curve of pure *T. laurifolia* DNA. By applying this approach, we were able to detect adulterations as low as 1 % (Fig. 2a).

### Identification of herbal species in commercial products

Constituent species in herbal products bought from markets in Thailand were investigated to assess the reliability of information regarding their ingredients, as the herbal products are often sold in processed forms. Ten herbal products labeled as ‘Rang Chuet’ were purchased and examined (Table 1). The HRM analysis using *rpoC* primers was then performed to identify the species in the products.

The examination of the HRM difference curve of all tested samples using *T. laurifolia* curve as baseline revealed that seven out of ten samples (COM3-7 and 9–10) produce curves in which the same as *T. laurifolia*’s with a 90% confidence interval, suggesting that the products contain *T. laurifolia* (Fig. 2b). The melting curve of one tested sample (COM8) was found between *T. laurifolia* and *C. spectabilis* lines, it could be indicated that the commercial COM8 was probably be admixture of *T. laurifolia* and *C. spectabilis* with around 3% of the toxic *C. spectabilis* in the product as show in Fig. 2b. However, we cannot rule out the possibility of the COM8 may actually not be contaminated with the toxic *C. spectabilis* but other species. In addition, the results of the analysis also reveal that the two remaining samples (COM1 and COM2) were much likely not contain any of the two ‘Rang Chuet’ species but some other species instead (Fig. 2b). In order to find contaminated or substituted species in COM1 and COM2, DNA barcoding is one of the best solutions. As can be seen from Newmaster *et al* [26] work, DNA barcoding was performed to detect the adulteration and substitution of herbal drugs and found that herbal products sold on the markets were contaminated or substituted with alternative plant species that are not listed on the labels as they are replaced entirely by powdered rice, wheat and soybean. Thus, DNA sequencing of *rbcL* region was carried out to identify species in these two products. The blast result showed that COM1 and COM2 have a similarity in their sequences to *Moringa oleifera* and *Andrographis paniculata*, respectively (Additional file 1: Table S1). The finding provides evidence that substitution in herbal products sold Thai local market is presented and this substitution could be a serious issue for consumers. Due to the fact that Bar-HRM has allowed us easily determine herbal species in processed products sold on the markets within 2 h. Bar-HRM method developed in this study therefore pose a potential to be a great tool in detection of adulteration and/or substitution in herbal products especially in processed forms.

## Conclusions

Several studies have shown that substitution of plant species occurs in herbal medicines, and this in turn poses a challenge to herbal authentication as adverse reactions might be due to substituted ingredients. Bar-HRM has proven to be a cost-effective and reliable method for the identification of species in this study of Thai medicinal plants. The hybrid method of DNA barcoding and High Resolution Melting is dependable, fast, and sensitive enough to distinguish between species. In this study, the tested products were traded as processed powder, which impedes conventional identification. Because of this processing it is almost impossible to identify which herbal species are present in products using morphological characters. The DNA extracted from all products tested yielded a specific amplification product with the designed *rpoC* Bar-HRM primers. The normalized HRM curves for the amplicons, from the two ‘Rang Chuet’ species (*T. laurifolia* and the toxic *C. spectabilis*) and ten herbal products, based on HRM analysis with barcode marker *rpoC* were easily distinguished, and seven of the ten tested samples were successfully assigned to the *T. laurifolia* species. However, three products were found to contain other plant species or admixture of the two ‘Rang Chuet’ species. Therefore, the developed method could be easily used for rapid and low-cost authentication of herbal products. Interestingly, designing primer for HRM analysis commonly depends on information in database but here even none of *rpo*C sequences of *Thunbergia* could be found on the database, the *rpo*C primer pair derived from DNA data of other random medicinal plant species is found to be work well in this analysis. It is demonstrated that this method has not only shown the great beneficial value for universality test which might be useful in other medicinal species but also flexibility using DNA region with limited data like *rpo*C.

## Additional file

Additional file 1: Table S1. Blast results of two commercial product *rbcL* sequences.
