# Supplementary material for: Fast and reliable detection of toxic Crotalaria spectabilis Roth. in Thunbergia laurifolia Lindl. herbal products using DNA barcoding coupled with HRM analysis
Source: BMC Complement Altern Med. 2015 May 30;15:162. doi: 10.1186/s12906-015-0692-6 (PMC4448308; doi:10.1186/s12906-015-0692-6)
Supplement: Additional file 1: Table S1. — Blast results of two commercial product rbcL sequences. [file 12906_2015_692_MOESM1_ESM.docx]

| **Sample** | **Abbreviation** | **Length (base)** | **NCBI/ BLAST Results** | | |
| --- | --- | --- | --- | --- | --- |
|  |  |  | **Ident** | **GenBank accession number** | **Species** |
| CNC  (GenBank accession number: KR046916) | COM1 | 489 | 99% | JX091931.1 | *Moringa oleifera* |
|  |  |  | 99% | L11359.2 | *Moringa oleifera* |
|  |  |  | 99% | X571866.1 | *Moringa oleifera* |
| TTC  (GenBank accession number: KR046917) | COM 2 | 487 | 99% | KF521880.1 | *Andrographis paniculata* |
|  |  |  | 99% | KF150644.2 | *Andrographis paniculata* |
|  |  |  | 99% | GQ436495.1 | *Andrographis paniculata* |

**Supplementary Data 1.**  Blast results of two commercial product *rbcL* sequences
